# Paraconsistent artificial neural networks and Alzheimer disease: a preliminary study

**DOI:** 10.1590/S1980-57642008DN10300004

**Published:** 2007

**Authors:** Jair Minoro Abe, Helder Frederico da Silva Lopes, Renato Anghinah

**Affiliations:** 1 Institute For Advanced Studies - University of São Paulo, Brazil.; 2 Graduate student of M edical School of University of São Paulo - Brazil.; 3 Reference Center of Behavioral Disturbances and Dementia (CEREDIC) of the Medical School of University of São Paulo, Brazil.

**Keywords:** EEG, Alzheimer disease, pattern recognition, artificial neural network, paraconsistent logic

## Abstract

EEG visual analysis has proved useful in aiding AD diagnosis, being indicated in some clinical protocols. However, such analysis is subject to the inherent imprecision of equipment, patient movements, electric registers, and individual variability of physician visual analysis.

**Objectives:**

To employ the Paraconsistent Artificial Neural Network to ascertain how to determine the degree of certainty of probable dementia diagnosis.

**Methods:**

Ten EEG records from patients with probable Alzheimer disease and ten controls were obtained during the awake state at rest. An EEG background between 8 Hz and 12 Hz was considered the normal pattern for patients, allowing a variance of 0.5 Hz.

**Results:**

The PANN was capable of accurately recognizing waves belonging to Alpha band with favorable evidence of 0.30 and contrary evidence of 0.19, while for waves not belonging to the Alpha pattern, an average favorable evidence of 0.19 and contrary evidence of 0.32 was obtained, indicating that PANN was efficient in recognizing Alpha waves in 80% of the cases evaluated in this study. Artificial Neural Networks – ANN – are well suited to tackle problems such as prediction and pattern recognition. The aim of this work was to recognize predetermined EEG patterns by using a new class of ANN, namely the Paraconsistent Artificial Neural Network – PANN, which is capable of handling uncertain, inconsistent and paracomplete information. An architecture is presented to serve as an auxiliary method in diagnosing Alzheimer disease.

**Conclusions:**

We believe the results show PANN to be a promising tool to handle EEG analysis, bearing in mind two considerations: the growing interest of experts in visual analysis of EEG, and the ability of PANN to deal directly with imprecise, inconsistent, and paracomplete data, thereby providing a valuable quantitative analysis.

## Abstract (pt)

A análise visual de EEG tem se mostrado útil na ajuda de diagnóstico de DA, sendo indicado em alguns protocolos clínicos. Porém, tal análise está sujeita à imprecisão inerente de equipamentos, movimentos do paciente, registros elétricos e variação individual da análise visual do médico.

**Objetivos:**

Utilizar a Rede Neural Artificial Paraconsistente para saber como determinar um grau de certeza no diagnóstico da doença de Alzheimer provável.

**Métodos:**

Dez pacientes com doença de Alzheimer provável e 10 controles foram submetidos ao registro de exames de EEG durante a vigília em repouso. Considerou-se como padrão normal de um paciente, a atividade de base entre 8,0 Hz a 12,0 Hz, permitindo uma variação de 0.5 Hz.

**Resultados:**

A RNAP foi capaz de reconhecer ondas que pertencem à banda Alfa como banda Alfa com evidência favorável de 0.30 e evidência contrária de 0.19, enquanto ondas não pertencentes ao padrão Alfa, foi obtido uma evidência favorável média de 0.19 e evidência contrária de 0.32, mostrando que a RNAP foi eficiente para reconhecer ondas Alfa, o que leva a uma concordância com o diagnóstico clínico de 80%.

**Conclusões:**

RNAP pode ser ferramenta promissora para manipular análise de EEG, tendo em mente ambas considerações: o interesse crescente de especialistas em análise visual de EEG e a capacidade da RNAP tratar diretamente dados imprecisos, inconsistentes e paracompletos, fornecendo uma interessante análise quantitativa.

Several studies on behavioral and cognitive neurology have been conducted to characterize dementias by means of biological and functional markers aimed at understanding the evolution of Alzheimer disease (AD), following its progression, as well as leading toward better diagnostic criteria for early detection of cognitive impairment.^1,2^

At present, there is no method able to determine a definitive diagnosis of dementia, where a combination of tests is needed to reach a probable diagnosis.^3^

The electroencephalogram (EEG) is a record of brain electrical signal activity, providing a space-time representation of synchronic postsynaptic potentials. The main generating sources of these electrical fields are most likely perpendicular in relation to the cortical surface, such as in the cortical pyramidal neurons.^4^ During the relaxed awake state, normal EEG in adults is predominantly composed by the alpha band frequency, which is generated by interactions of the slum-cortical cortical and thalamocortical systems.^5,6^

Several studies have shown that EEG visual analysis is useful in aiding AD diagnosis, being indicated in some clinical protocols.^3,4^

The most common finding in EEG visual analysis is the slowing background of the brain electrical activity compounds regarding delta and theta rhythms, and the decreasing or absence of the alpha rhythm. However, these findings are more common in moderate and advanced stages of AD.^3^

The majority of theories and techniques available for quantitative EEG analysis are based on classical logic and so cannot adequately such sets of information, at least directly. Although several theories have been developed in order to overcome these limitations, e.g. Fuzzy set theory, Rough theory, non-monotonic reasoning, among others, they cannot deal with inconsistencies and paracompleteness. Thus, we need a new kind of logic to deal with uncertain, inconsistent and paracomplete data.^7,8^

The Artificial Neural Network – ANN – can be described as a computational system consisting of a set of highly interconnected processing elements, called artificial neurons, which process information as a response to external stimuli. An artificial neuron is a simplistic representation that emulates the signal integration and threshold firing behavior of biological neurons by means of mathematical structures. Like their biological counterparts, artificial neurons are bound together by connections that determine the flow of information between peer neurons. Stimuli are transmitted from one processing element to another via synapses or interconnections, which can be excitatory or inhibitory.^9,10^

The advantage of neural networks over conventional programming lies in their ability to solve problems that do not have an algorithmic solution or where the available solution is too complex to be found.^11^ Neural networks are well suited to tackle problems that people are good at solving, such as prediction and pattern recognition. Neural networks have been applied within the medical domain for clinical diagnosis,^15^ image analysis and interpretation,^12,13^ signal analysis and interpretation, and drug development.^14^

Therefore, ANN constitutes an interesting tool for EEG qualitative analysis. On the other hand, in EEG analysis we are faced with imprecise, inconsistent and paracomplete data. In order to manipulate this information directly, some interesting theories have been proposed recently: Fuzzy sets, Rough sets, among others.

In this study we employed a new kind of ANN based on Paraconsistent Annotated Evidential Logic Eτ, which is capable of manipulating imprecise, inconsistent and paracomplete data in order to make a first study of the recognition of EEG standards with the aim of using this further in AD diagnosis.

Paraconsistent Artificial Neural Networks – PANN is a new artificial neural network that will be presented briefly below.

The atomic formulas of the logic Eτ are of the type *p*_(µ,λ)_, where (µ,λ) ∈ [0, 1]^2^ and [0, 1] is the real unitary interval (*p* denotes a propositional variable). *p*_(µ,λ)_ can be intuitively read: “It is assumed that p’s favorable evidence is µ and contrary evidence is λ” Thus,

- p_(1.0, 0.0)_ can be read as a true proposition.
- p_(0.0, 1.0)_ can be read as a false proposition.
- p_(1.0, 1.0)_ can be read as an inconsistent proposition.
- p_(0.0, 0.0)_ can be read as a paracomplete (unknown) proposition.
- p_(0.5, 0.5)_ can be read as an indefinite proposition.

We introduce the following concepts (all considerations are made with 0 ≤ µ, λ ≤ 1):Uncertainty Degree: G_un_(µ,λ) = µ+λ–1; Certainty Degree: G_ce_(µ,λ) = µ–λ; an order relation is defined on [0, 1]^2^: (µ_1_, λ_1_) = (µ_2_, λ_2_) ⇔ µ_1_ ≤ µ_2_ and λ_1_ ≤ λ_2_, constituting a lattice that will be symbolized by τ.

With the uncertainty and certainty degrees we can achieve the following 12 output states: *extreme states* that are, False, True, Inconsistent and Paracomplete, and *non-extreme states.*

Some additional control values are:

- V_cic_=maximum value of uncertainty control=Ft_ct_
- V_cve_=maximum value of certainty control=Ft_ce_
- V_cpa_=minimum value of uncertainty control= –Ft_ct_
- V_cfa_=minimum value of certainty control= –Ft_ce_

For the discussion in the present paper we have used: Ft_ct_=Ft_ce_= ½.

All states are represented in Figure 1.

**Figure 1:**
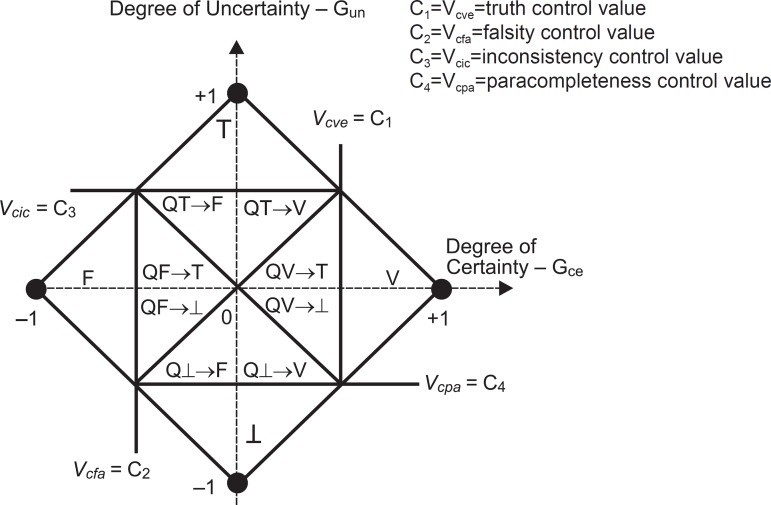
The figure displays the output regions of the lattice, constituting the decision-making of the inputs. In this lattice we have 12 output states: extreme and non-extreme states. See Table 1 for symbology.

**Table 1:** Extreme and non-extreme states.

Extreme states	Symbol	Non-extreme states	Symbol
True	V	Quasi-true tending to Inconsistent	QV→T
False	F	Quasi-true tending to Paracomplete	QV→⊥
Inconsistent	T	Quasi-false tending to Inconsistent	QF→T
Paracomplete	⊥	Quasi-false tending to Paracomplete	QF→⊥
		Quasi-inconsistent tending to True	QT→V
		Quasi-inconsistent tending to False	QT→F
		Quasi-paracomplete tending to True	Q⊥→V
		Quasi-paracomplete tending to False	Q⊥→F

In the PANN the main aim is to ascertain how to determine the certainty degree concerning a proposition, i.e.

if it is False or True. To this end, we take into account the certainty degree G_ce_. The uncertainty degree G_un_ indicates the ‘measure’ of the inconsistency or paracompleteness. If the certainty degree is low or the uncertainty degree is high, it generates an indefinition.

Using the concepts of *basic* Paraconsistent Artificial Neural Cell – PANC, we can obtain the family of PANC considered in this work, as described in Table 2.

**Table 2:** Paraconsistent artificial neural cells.

PANC	Inputs	Calculations	Output
Analytic connection - PANC_ac_	µ, λ, Ft_ct_, Ft_ce_	λc=1 - λ , G_un_, G_ce_, µr=(G_ce_ + 1)/2	If |G_ce_| > Ft_ce_ then S_1_ = µr and S_2_ = 0;
			If |G_un_| > Ft_ct_ and |G_un_| > | G_ce_| then S_1_ = µ_r_ and S_2_ = |G_un_|, if not S_1_ = ½
			and S_2_ = 0
Maximization - PANC_max_	µ, λ	none	If µ > λ, then S_1_ = µ, if not S_1_ = λ
Minimization - PANC_min_	µ, λ	none	If µ < λ, then S_1_ = µ, if not S_1_ = λ

## Methods

We analyzed 10 controls and 10 AD EEGs records, during the awake state at rest, with subjects closing their eyes.

We used electrodes placed according to the 10-20 international system, a 32 EEG channel EMSA device, with 200Hz sample frequency.

The process of wave analysis by PANN consists previously of:

- Data capturing
- Adaptation of the values for screen examination
- Elimination of the negative cycle
- Normalization of the values for PANN analysis

It is worth observing that the process above does not allow the loss of any essential wave characteristics for our analysis.

The capturing of the data is obtained from magnetic files (suitable software for physical capture of the signals) or manually (TXT files - American National Standard Code for Information Interchange).

As the actual EEG examination values can vary highly, in a module, somewhere between 10 µV to 1500 µV, we proceed with normalization of the values to between 100 µV and –100 µV by a simple linear conversion, to facilitate the processing and to visualize on the screen:

$$x=\frac{100.a}{m}$$

where *m* is the maximum value of the exam; a is the current value of the exam. Therefore, *x* is the current normalized value.

### Elimination of the negative cycle

The minimum value of the exam is taken as a zero value and the remaining values are translated proportionally.

### Data analysis, expert system, and wave morphology

In analyzing EEG signals, one important aspect to take into account is the morphological aspect. To perform this task, it is valuable to build one very simple Expert System, which allows “abnormalities” to be verified, such as spikes and artifacts. Also, it analyses the signal behavior, verifying which band it belongs to (delta, theta, alpha and beta).

### Morphological analysis

The process of the morphological analysis of a wave is performed by comparing with a certain set of wave patterns (considered normal). A wave is associated to a vector (finite sequence of natural numbers x_i_) through digital sampling. This vector characterizes a wave pattern and is registered by the PANN. Thus, new waves are compared, allowing their recognition or otherwise. For the sake of completeness, we show some basic aspects of how PANN operates. Let us take three vectors: V_1_=(8, 5, 4, 6, 1); V_2_= (8, 6, 4, 6, 5); V_3_=(8, 2, 4, 6, 9). The favorable evidence is calculated as follows: given a pair of vectors, we take ‘1’ for equal elements and ‘0’ for different elements, and calculate their percentage.

- Comparing V_2_ with V_1_: 1+0+1+1+0=3; in percentage: (3/5)*100=60%.
- Comparing V_3_ with V_1_: 1+0+1+1+0=3; in percentage: (3/5)*100=60%.

The contrary evidence is the weighted addition of the differences between the different elements, in the module:

- Comparing V_2_ with V_1_=0+1/10+0+0+4/10=(5/10)/5 =10%.
- Comparing V_3_ with V_1_=0+3/10+0+0+8/10=(11/10)/5 =22%.

Therefore, we can state that V_2_ is ‘more similar’ to V_1_ than V_3_. We use a PANN to recognize this system technique.

Also, the PANN is capable of adjusting its own recognizing factor and propagate to other layers, improving both ‘proximity’ level and ‘recognizing’ level, while also providing the approximate frequency of an analyzed wave.

For the purposes of this study, the favorable evidence was obtained by counting the wave peaks, i.e., the closer to the peak quantity, the greater the degree of favorable evidence:

EF=1–((|bd – vt|)/(bd + vt)), where:

- Vt=number of wave peaks of the exam
- Bd=number of the wave peaks being compared (pattern stored in the database)

Each peak is a 1 Hz morphological approximation; so a wave with 8 peaks is classified as 8 Hz wave (Alpha band).

### Data analysis

*Expert system for detecting the diminishing average frequency level* – An expert system verifies the average frequency level of Alpha waves and compares them with a fixed external one (external parameter wave).

Such external parameter can be, for instance, the average frequency of a population or the average frequency of the last exam of the patient. This system also generates two outputs: favorable evidence µ (normalized values ranging from 0 (corresponds to 100% – or greater frequency loss) to 1 (which corresponds to 0% of frequency loss) and contrary evidence λ (=1–µ).

The population pattern used in this work is 10 Hz (6).

*Expert system for Alpha band concentration* – This expert system is utilized for Alpha band concentration in the exam. For this, we consider the quotient of the sum of fast Alpha and Beta waves over slow Delta and Theta waves, i.e., (Alpha + Beta) / (Delta + Theta).

This expert system generates two outputs:

- Favorable evidence µ: fast waves/(fast waves + slow waves).
- Contrary evidence: λ=1–µ.

*Expert system for Theta band concentration* – This expert system is utilized for Delta band concentration in the exam. We consider (slow waves Delta + slow waves Theta) / (fast waves Alpha + fast waves Beta).

This expert system generates two outputs:

- Favorable evidence µ: 1–((slow waves)/(fast waves + slow waves)).
- Contrary evidence: λ=1–µ.

### Data analysis

When analyzing information from sources, we may encounter contradictory, fuzzy or paracomplete data. However, a decision can still be reached. For this, suppose that we have three items of information PA, PB, and PC, where PA and PB are being analyzed. If we cannot decide with this expert information, we take into account the third PC in the following way.

The first layer is composed of three analytical PANC connections: C1, C2, and C3 whose signals are analyzed by means of the Basic Structural Equation: BSE: S=(µ–(1–λ) +1) / 2 resulting in the output signals SA, SB, and SC.

In the internal layers, the cells C4 and C6 constitute the Maximization Neural Unit (it takes the maximum value SG among output values SA, SB, and SC) and the cells C5 and C7, the Minimization Neural Unit (which takes the minimum value SE among output values SA, SB, and SC).

Another way to define the interpretation of the analysis is to use the resultant value (_r_) and complements it; this generates a complemented resultant value (λ_r_). In this manner, we acquire resultant favorable evidence (µ_r_) and resultant contrary evidence (λ_r_), as exemplified below:

We applied this methodology in two subjects (blind study), one of them normal and the other with AD, and the method was able to distinguish both, classifying the normal subject in the normal standard of waves and the AD subject under the abnormal model.

## Results

The proposed method was tested on 10 EEGs and the system correctly classified normal subjects at a rate of 80% with 20% as false-positive (Table 3).

**Table 3:** Test with normal patients.

Casuistic	Patient	FE	CE	Mean	Diagnosis
7601	JS	0.4813	0.1404	6.9184	1
7701	RKG	0.4813	0.0712	8.475	2
5401	EC	0.4959	0.1377	7.025	2
7801	JIS	0.5191	0.0603	8.5	1
6501	LANG	0.5207	0.0548	8.425	1
7101	JTBT	0.5419	0.0594	8.6	1
7201	OTWNV	0.5896	0.0301	8.4	1
1202	RA	0.8162	0.0613	10.2	1
2102	DYT	0.8546	0.0485	18.825	1
1802	DO	0.8818	0.0394	10.15	1

FE, favorable evidence; CE, contrary evidence; 1, normal patient; 2, probable AD patient.

In the following study, a further 10 EEGs were tested and the system correctly classified AD cases at a rate of 80% with 20% false-negative, as shown in Table 4.

**Table 4:** Test with non-normal patients.

Casuistic	Patient	FE	CE	Mean	Diagnosis
4101	MTRS	0.3311	0.0596	7.55	2
6001	EGT	0.4373	0.2072	5.921	2
7901	AMNT	0.6851	0.0800	9.625	1
5701	ABC	0.7398	0.0584	9.575	2
2203	JPNF	0.1204	0.1185	6.175	2
6201	ESE	0.1623	0.1159	7.55	2
6301	MF	0.1865	0.1028	7.475	2
7301	AOFFS	0.2332	0.0856	7.45	1
5501	TMOG	0.2352	0.1551	6.15	2
6401	RRS	0.2564	0.1721	6.3	2

FE, favorable evidence; CE, contrary evidence; 1, normal patient; 2, probable AD patient.

## Discussion

For this preliminary test the normal pattern of a patient was considered as waves from 8 Hz to 12 Hz, allowing a variance of 0.5 Hz, thus representing the Alpha band. We stored 9 positive cycle wave patterns plus 9 negative cycle wave patterns. In this test periods were selected containing characteristics of the Alpha band, totaling 10 seconds. The PANN was capable of recognizing waves belonging to the Alpha band with favorable evidence of 0.30 and contrary evidence of 0.19, while for waves not belonging to the Alpha pattern, an average favorable evidence of 0.19 and contrary evidence of 0.32 was achieved, demonstrating that PANN was efficient in recognizing Alpha waves.

**Figure 5:**
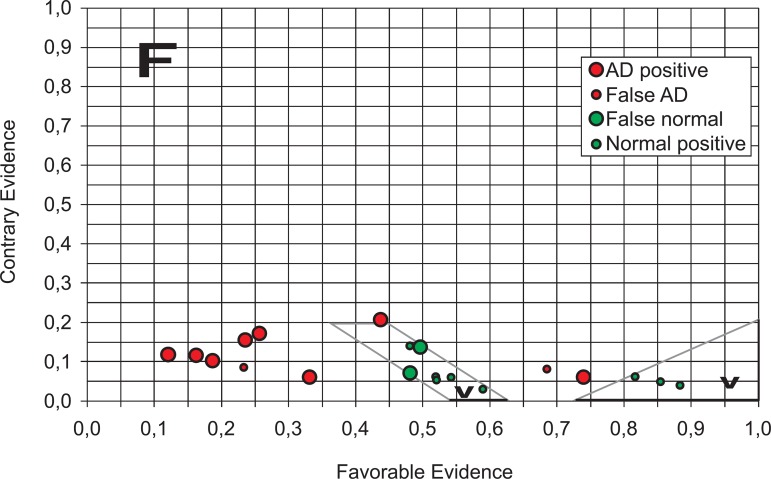
Final lattice of diagnosis decision states – Normal x Probable AD patients. We can observe two groups of normality: those non AD patients with Alpha concentration higher or equal to the average rate population (triangle of right side) and remaning non AD patients with Alpha concentration lower than average population rate. F, false output state; V, true output state.

The lattice in Figure 5 depicts two true state regions. This can be indicative of the existence of two groups of normality: non AD patients with Alpha concentration higher or equal to the average population rate (triangle to right side) and the remaining subjects which are non AD patients with Alpha concentration lower than the average population rate. The same observation seems applicable to the probable AD patients. A more complete study is currently underway the results of which are set to appear in forthcoming papers.

## Figures and Tables

**Figure 2:**
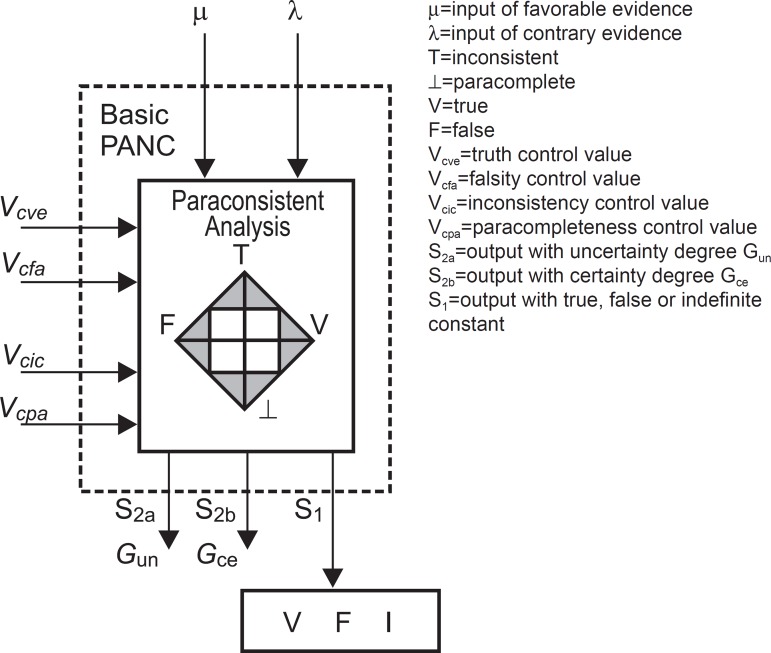
Basic cell of PANN. The resulting certainty degree Gce is obtained as follows: If: V_cfa_ ≤ G_un_ ≤ V_cve_ or V_cic_ ≤ G_un_ ≤ V_cpa_ ⇒ G_ce_ = Indefinition For: V_cpa_ ≤ G_un_ ≤ V_cic_ If: G_un_ ≤ V_cfa_ ⇒ G_ce_ = False with degree G_un_ V_cic_ ≤ G_un_ ⇒ G_ce_ = True with degree G_un_ A Paraconsistent Artificial Neural Cell – PANC – is called basic PANC when a given pair (µ, λ) is used as input, and resulting as output: G_un_=resulting uncertainty degree, G_ce_=resulting certainty degree, and X=constant of Indefinition.

**Figure 3:**
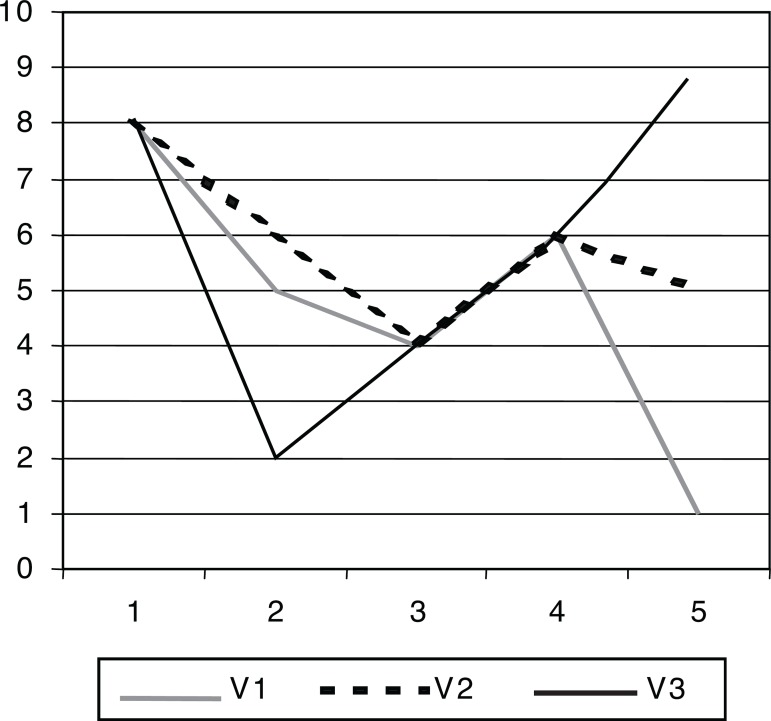
Comparison of the vectors. Taking as basis the vector V_1_, visually we can observe that vector V_2_ is ‘more similar’ to V_1_ than V_3_. We use a PANN to recognize this technical system.

**Figure 4:**
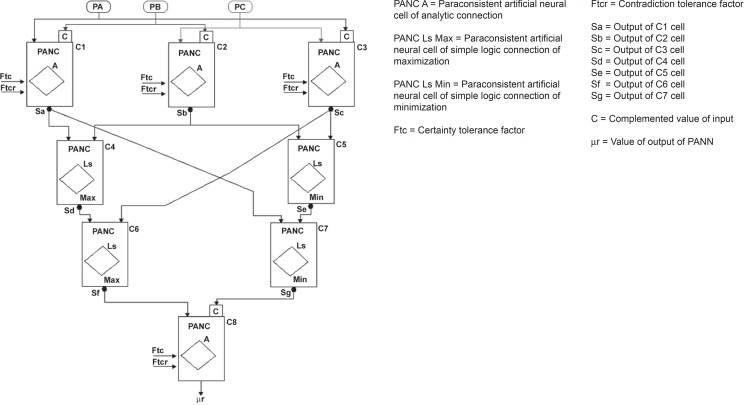
A decision-making architecture for global analysis. Three expert systems operate: PA, for detecting the diminishing average frequency level; PB, for Alpha band concentration, and PC, for Theta band concentration. C_1_–PANC which processes input data of PA and PB C_2_–PANC which processes input data of PB and PC C_3_–PANC which processes input data of PC and PA C_1_, C_2_, and C_3_ constitute the 1^st^ layer of the architecture C_4_–PANC which calculates the maximum evidence value between cells C_1_ and C_2_ C_5_–PANC which calculates the minimum evidence value between cells C_2_ and C_3_ C_4_ and C_5_ constitute the 2^nd^ layer of the architecture C_6_–PANC which calculates the maximum evidence value between cells C_4_ and C_3_ C_7_–PANC which calculates the minimum evidence value between cells C_1_ and C_5_ C_6_ and C_7_ constitute the 3^rd^ layer of the architecture C_8_ analyzes the experts PA, PB, and PC and gives the resulting decision value
